# Timetable for oral prevention in childhood—a current opinion

**DOI:** 10.1186/s40510-015-0098-5

**Published:** 2015-08-26

**Authors:** Paddy Fleming

**Affiliations:** Dublin Dental University Hospital, Trinity College Dublin, Dublin 2, Ireland; Dental Department, Our Lady’s Children’s Hospital Crumlin, Dublin 12, Ireland

**Keywords:** Oral health, Children, Dental caries, Prevention, Toothbrushing, Toothpaste, Fluoride, Infant feeding, Early childhood caries

## Abstract

Dental caries in young children remains a public health problem particularly for children whose families are socioeconomically deprived. A child's first dental visit should be at approximately 12 months of age and this should facilitate the provision of anticipatory guidance concerning oral health and dental development to the child's parents/guardians. Compliance with dietary advice is of key importance and motivational interviewing shows promise in relation to parents adopting good oral health practices for their children. Twice daily toothbrushing using toothpaste that contains in the range of 1000- 1500ppmF is a most important preventive measure. It is important to use a minimal amount of toothpaste, insure that it is not swallowed, have parental or adult supervision during toothbrushing and avoid rinsing with water following brushing with toothpaste. The professional application of topical fluoride varnish twice yearly is a proven caries preventative measure. The application of pit and fissure sealants to teeth with deep pits and fissures is recommended.

## Review

### Introduction

Health may be defined as a state of complete physical, mental and social wellbeing and not merely the absence of disease [1]. Oral health is an essential component of general health and influences a person’s quality of life. Children should enjoy a high standard of health, including oral health, but as children, they are dependent on adults for this. The dental health profession has a responsibility to advocate for optimum oral health for all children. A major component of this advocacy should concern the prevention of oral disease—the most common chronic disease in children being dental caries—and appropriate management of dental caries that has not been prevented.

### The problem of dental caries in young children

Dental caries is the most common chronic disease of childhood and may affect a child’s quality of life due to various factors including pain, infection, sleep loss, absence from school with compromised education, poor mastication, compromised appearance, and inadequate nutrition, growth and development. In industrialised countries, dental caries remains a significant public health problem. For example, the recently published national epidemiological study on the oral health of children in England, Wales and Northern Ireland reported that 31 % of 5-year-olds had obvious decay experience [2]. The average number of teeth affected (dmft) in those 5 year olds with decay was 3.0. A third (34 %) of 12-year-olds and 46 % of 15-year-olds had dental caries or had treatment provided for caries of their permanent teeth. The mean dmft in 12-year-olds with dental caries experience was 2.5. There was a strong and consistent relationship between levels of deprivation and severity or extent of decay—thus, there was a considerable burden of disease in socioeconomically deprived children who had dental caries. Similar results have been reported from epidemiological studies in other countries. In addition, because it is not feasible to record bitewing radiographs in large epidemiological studies, the number of carious teeth is likely an underestimate in many children. For very young children, data from the National Health and Nutrition Examination Survey (NHANES) in the USA, which was collected from 2011 to 2012, showed that 23 % of pre-school children had experienced dental caries [3].

Dental treatment of carious primary teeth in children is very costly with further dental treatment being subsequently required in many young children who have had comprehensive dental treatment provided [4]. Dental caries or its treatment in the primary dentition is a strong predictor for future development of dental caries in the primary, mixed and permanent dentitions. It is therefore recommended that parents and parents-to-be should be informed, encouraged and hopefully motivated to prevent the development of dental caries in their infants and young children.

### Expectant mothers

Dentists may be invited to present information concerning oral health to expectant mothers during prenatal classes. Paediatric dentists need to be aware that oral pregnancy-related changes are most frequent and most marked in the gingival tissues. Pre-existing gingivitis, due to hormonal changes in the gingival vasculature, may become more severe and manifest as ‘pregnancy gingivitis’ with dark red, swollen, gingivae that bleed easily. A relationship between poor maternal, periodontal health and pre-term delivery has been reported and continues to be investigated. An increase in salivary levels of mutans streptococci may occur, possibly associated with increased frequency of snacking because of metabolic demands of pregnancy. The paediatric dentist should promote good maternal oral health during pregnancy and after delivery, with an emphasis on a good diet and good oral hygiene practices [5].

Information concerning infant oral growth and development, including eruption of primary teeth, should be provided. Advice should also be given concerning infant feeding and its potential relationship with early childhood caries. Salivary mutans streptococci may be transmitted from mother to infant and young children, during feeding, so advice should be given to avoid sharing feeding utensils when feeding infants and young children [6].

The presence of a dental professional at prenatal classes is likely to increase awareness within the general population, amongst medical colleagues and amongst opinion makers and decision makers of the importance of oral and dental health as part of general health. When an expectant mother attends a general dentist for her own dental care, preventive advice concerning future infant and child oral health should be given by the dentist or by a member of the dental team to the expectant mother as well as providing her dental treatment.

### The first dental visit by a child’s first birthday

It is recommended that a baby’s first dental visit should be at approximately 12 months of age. A ‘baby visit’ is a happy occasion in a dental practice with all staff naturally delighted to see a little infant attend for an oral examination.

The oral examination is best undertaken using the ‘knee-to-knee’ approach, with the infant lying supine, supported by the parent on her/his lap, and the infant’s head lying on the dentist’s lap. A good light source, such as the dental operatory light, is essential for examination of the oral soft tissues, alveolar ridges, palate and any erupted or erupting teeth. The teeth should be dried with gauze or with a cotton roll or cotton bud in order to visualise any developmental anomalies such as enamel opacities or hypoplasia and also to detect indicators of dental caries such as biofilm (plaque) deposits, gingivitis, demineralised enamel or cavitation of teeth.

Anticipatory guidance in relation to a child’s oral health is a term used to anticipate the next stage of growth and development of an infant or child and give guidance to parents concerning what they may expect and what practices they might undertake to benefit their infant’s or child’s oral health. This visit is a wonderful opportunity for the dentist and team to provide such guidance to the mother and/or parents and also allows the opportunity to discuss prevention of early childhood caries [7].

### Toothbrushing

Parents should use an appropriately sized toothbrush to commence tooth brushing as soon as their infant’s first tooth erupts. Toothpastes that contain fluoride in the range 1000 to 1500 ppmF are effective but because of the small size of an infant and very young child, it is important that the paste is not ingested as this could put the developing teeth, including the permanent anterior teeth, at risk of fluorosis. Therefore, only a smear of toothpaste, 0.1 ml volume, the size of an uncooked grain of rice, should be used, making sure that none is swallowed.

Once a child reaches 3 years of age, the amount of toothpaste may be increased to the size of a small pea, 0.25 ml volume, but again it is important that this is not swallowed and toothbrushing should continue to be supervised by an adult throughout childhood. Children should be encouraged to spit out excess paste but not to rinse with water following brushing with toothpaste [8, 9].

High dose fluoride toothpastes, containing 2800 ppmF, are available on prescription in the UK and are recommended for twice daily use in children aged 10 years and over who are assessed to be at high risk of developing caries [9].

It is recommended that toothbrushing should take place last thing at night before bedtime and on at least one other occasion during the day. The duration of toothbrushing should exceed 1 min on each occasion, and eating directly after brushing should be avoided [8].

The gingivae associated with primary teeth in healthy infants and young children should be clean and pink coloured even if the teeth are not brushed. If biofilm (plaque) deposits and/or gingivitis are present in a healthy infant or young child, the clinician must suspect the presence of high numbers of mutans streptococci associated with frequent ingestion of sugary drinks, juices, diluted juices or sugary foods and classify the child as at high caries risk.

### Feeding practices in infants and young children

An infant or young child should never sleep or nap with a feeding bottle or a feeding cup containing formula feed, fortified feed, milk or sweetened fluid as this habit will likely lead to severe early childhood caries. Some parents find that the use of a bottle containing only water is helpful in weaning their child from use of a feeding bottle. Sweetened fluids, juices or diluted juices should not be taken at any time from a bottle. Juice may be taken from a feeding cup as part of a meal but at all other times, including at most meals, water or milk should be the drink of choice.

Breastfeeding is encouraged as the ideal form of feeding for young infants. However, in some instances, older infants with erupted teeth and some very young children will breastfeed frequently on demand throughout the night—putting their erupted teeth at high risk of developing caries. In such circumstances, there should be a discussion with the child’s mother explaining the link between prolonged breastfeeding during sleep, when there is little or no salivary flow, in an infant or young child with teeth, and early childhood caries. If feasible, breastfeeding in older infants with erupted teeth and in very young children should be limited to short periods of time when they are awake.

The frequent use of juices, diluted juices and other sweetened fluids is often associated with early childhood caries, and dental erosion may also occur. The infant’s mother/parents should be advised to follow their doctor’s or paediatrician’s advice and introduce savoury rather than sweet foods to their developing young child’s diet, plan a practical balanced diet, and use water and milk as the main drinks. In older children and in adults, the frequent consumption of sweet drinks and juices may be associated with the development of unhealthy weight gain, obesity, dental caries and erosion [10, 11].

Sweet foods including honey and dried fruit, confectionary bars and biscuits should be limited to special occasions only. Sweet drinks and juices, including diluted juices, have added sugars and their use should be restricted, with water or milk being the main drink during the day and with most meals. The current guideline of the World Health Organization, in relation to dietary intake of sugars in both adults and children, strongly recommends a reduction in the intake of free sugars to less than 10 % of total energy intake [12]. Free sugars include monosaccharides and disaccharides added to foods and beverages by the manufacturer, cook or consumer, and sugars naturally present in honey, syrups, fruit juices and fruit juice concentrates.

Parental education and compliance with preventive advice concerning feeding may prevent the development of early childhood caries, allow remineralisation of any demineralised lesions and arrest the progress of cavitated lesions that may subsequently be treated restoratively or surgically. Motivational interviewing (MI) is a relatively recently developed, goal-oriented, client-centred counselling style for eliciting behaviour change by helping clients to explore and resolve ambivilance. Compared with non-directive counselling, it is more focused and goal-directed. There have been encouraging results from numerous studies that have used parent-involved motivational interviewing to improve paediatric oral health behaviour, but more studies are required to see if MI may predictably reduce dental caries in young children [13].

### Frequency of dental visits for young children

Annual review appointments may be arranged for those infants who are judged to be at low risk of developing caries, and reassessment of caries risk should be made at each review appointment. If an infant or young child is judged to be at high risk of developing caries, then review appointments at four to six monthly intervals should be arranged to provide professional preventive treatment, including the application of topical fluoride varnish on at least a six-monthly basis, and operative dental care as appropriate. Once a child has experienced dental caries, then frequent review appointments will be necessary.

When assessing caries risk in a child, the following factors should be considered: clinical evidence of previous disease, dietary habits, especially frequency of sugary food and drink consumption, social history especially socioeconomic status, use of fluoride, plaque control, saliva and medical history [9]. There are various programmes/tools available to assess caries risk, but provided multiple factors are taken into account, it does not appear that one system is significantly better than another in predicting future caries activity.

Health care workers other than dentists, such as public health nurses, paediatricians and nursery school teachers who have frequent early contacts with infants and very young children, may also undertake caries risk assessments and refer children judged to be at high risk of developing caries to dental practitioners or specialist paediatric dentists. Pubic health programmes for very young children with supervised nursery school toothbrushing have resulted in a reduction in dental caries in the west of Scotland [14], and preventive measures, including twice yearly topical application of fluoride varnish by paediatricians, have been valuable for children in primary care settings in the USA [15].

Regular review visits allow the dentist and team to provide ongoing appropriate anticipatory guidance and dental health education to the parent/s or carers of the young child. There are also likely positive psychological benefits for the child who attends for frequent non-invasive dental visits, in keeping with the latent inhibition hypothesis proposed by Davey [16]. Frequent non-threatening visits are thus likely to protect the young child from developing anxiety or fear following a potential future invasive dental procedure such as a dental extraction [17].

### Preventive dental procedures

Early diagnosis and intervention is necessary to prevent the development and rapid progression of dental caries in children. The professional application of topical fluoride varnish (22,600 ppmF) twice yearly in children has been shown to significantly reduce caries in primary and in permanent teeth, and the current SIGN guideline concerning dental interventions to prevent caries in children recommends this for all children in Scotland [9]. This recommendation may not be possible to implement for all children in other countries, but it should be implemented for children who are at high risk for dental caries.

In high-risk caries children, glass ionomer or resin pit and fissure sealants may be placed on the occlusal surfaces of primary molars that have deep fissure patterns—these sealants may be replaced at review visits if the sealants are no longer present and if the teeth are not carious.

Erupting or recently erupted permanent first molars that have deep pits and fissures are at risk of developing caries because of their anatomical configuration. Pit and fissure sealants, ideally resin sealants, should be used to prevent the development of carious lesions in such teeth in all children and should be placed as soon as possible following eruption of these teeth. In some children, it may not be feasible to achieve adequate moisture control for placement of resin sealants on recently erupted permanent first molars. In such cases, the placement of glass ionomer sealants, as an interim measure until the child is older, may be undertaken, and these sealants may be replaced if noted to be no longer present but without caries, at review visits [18, 19]. Resin sealants, when placed, also need to be evaluated at review visits and if no longer present will need to be replaced unless there is a carious cavity that requires restoration. The erupting permanent second molars in older children should be treated in a similar preventive manner.

The maxillary permanent incisors, particularly the lateral incisors, may have deep palatal pits. Periapical radiographs and sensibility tests should be recorded if dens invaginatus lesions are suspected. Fissure sealants using standard unfilled resin or using flowable composite should be undertaken as soon as possible after eruption because the dental hard tissues at the bottom of the invagination may be very porous and close to the pulp. Further restorative and/or pulpal therapy may be undertaken later.

Examination of the interproximal surfaces of primary and permanent teeth that have closed contacts is best undertaken with bitewing radiographs. In very young children or in children who find it difficult to accept horizontal bitewing radiographs, the placement of the smallest size dental film (size 0) vertically with the child biting on a holder allows for more space as the teeth are not in occlusion and the film does not extend as far posteriorly as the traditional horizontally positioned films. The frequency of recording bitewing radiographs is determined by the caries risk—if this is high, then bitewing radiographs may need to be recorded as frequently as on a yearly basis while children with a low caries risk may have bitewings every 3–4 years as the dentition develops at 5, at 8–9 and at 12–14 years of age. The EAPD guidelines on dental radiography in children give very helpful guidance in relation to this [20].

Early diagnosis allows conservative tooth preparations for restoration of carious cavities with composite resin or glass ionomer tooth coloured materials. If aesthetics is not a concern, preformed metal crowns may be used with traditional tooth preparation and rubber dam isolation under local analgesia or the Hall technique may be used with larger sized crowns selected because there is no tooth preparation. Multi-surface or very extensive lesions in primary molars are best treated with preformed metal crowns because recurrent caries of teeth restored with these crowns do not occur and these restorations will last, without requiring further treatment, until the primary molars spontaneously exfoliate.

## Conclusions

Dental caries in young children remains a public health problem particularly for children whose families are socioeconomically deprived. A child’s first dental visit should be at approximately 12 months of age, and this should facilitate the provision of anticipatory guidance concerning oral health and dental development to the child’s parents/guardians. Compliance with dietary advice is of key importance, and motivational interviewing shows promise in relation to parents adopting good oral health practices for their children.

Twice daily toothbrushing using toothpaste that contains fluoride in the range of 1000–1500 ppmF is a most important preventive measure. It is important to use a minimal amount of toothpaste, insure that it is not swallowed, have parental or adult supervision during toothbrushing and avoid rinsing with water following brushing with toothpaste. The professional application of topical fluoride varnish twice yearly is a proven caries preventative measure.

The application of pit and fissure sealants to teeth with deep pits and fissures is recommended. Glass ionomer may be used as a transitional sealant material if moisture control is not feasible in a young child or in a partially erupted tooth. A resin sealant may be applied at a subsequent visit when moisture control is possible.
